# Hydro-Mechanical Behaviour of an Unbound Granular Base Course Material Used in Low Traffic Pavements

**DOI:** 10.3390/ma13040852

**Published:** 2020-02-13

**Authors:** Peng Jing, Cyrille Chazallon

**Affiliations:** 1 College of Metropolitan Transportation, Beijing University of Technology, Beijing 100124, China; 2 ICube, UMR7357, CNRS, Université de Strasbourg, INSA de Strasbourg, 24 Boulevard de la Victoire, 67084 Strasbourg CEDEX, France; cyrille.chazallon@insa-strasbourg.fr

**Keywords:** unbound granular material, repeated load triaxial test, permanent/resilient deformation, prediction model, water content

## Abstract

This paper deals with the mechanical behaviour, especially the permanent deformation and resilient deformation of an unbound granular material (UGM) from Bréfauchet quarry which is used as base layer material in low traffic pavements for full-scale tests at IFSTTAR in France. Medium-scale repeated load triaxial tests (RLTT) are carried out at different water contents and the results show that both permanent and resilient deformations increase with water content. Besides, two techniques of fixing the sensors in materials with large particles for RLTTs are proposed and compared with each other. The results suggest that the tube method is more suitable for the UGM for an accurate measurement and a good adaptability is obtained during the RLTT. Based on the test results of UGM Bréfauchet, the modelling work is performed with improved models used previously for a sandy material. It appears that both the permanent and resilient behaviours of different unbound granular materials can be well captured by the proposed equations considering the effects of water content and anisotropy. This study is helpful to understand the evolution of permanent and resilient deformation in different granular layers, especially for the base layer, in low traffic pavements. The verified models can be used for other similar granular materials and this will lead to reducing the number of tests required to predict the deformation behaviour of granular materials.

## 1. Introduction

Flexible road pavements consist in upper asphalt layers over one or more unbound granular layers which are together compacted over a suitable soil subgrade. They represent approximately 60% of the road network in France.

Unbound granular materials (UGM) are continuously graded granular materials, consisting in general of crushed rock particles. They usually contain a certain amount of fines (typically 4% to 10%) and water and they are generally partially saturated. In flexible pavements, the response of unbound granular materials gives rise in resilient and permanent deformations when subjected to repeated loading. As it is well known, resilient deformations are related to the stiffness characteristics of the material that should be sufficiently high in order to avoid the fatigue cracking of overlying asphalt layers. On the other hand, the gradual accumulation of permanent deformations, although they are very small during each loading cycle, could lead to large ruts at the top of the structure due to excessive rutting [1,2,3]. As a result, well understanding of the UGM mechanical behaviour (especially the permanent and resilient deformation behaviour) is very helpful for the pavement design and maintenance.

In recent years, the effect of moisture content on deformation behaviour, which was equally important, has been studied by many researchers [4,5,6,7,8,9,10,11,12,13]. These researches showed that the permanent or resilient deformations increased with water content and different prediction models were proposed to evaluate the deformation behaviour of unbound granular materials. Especially, based on a long-term research on a subgrade material—Missillac sand (Missillac sand is an alluvium sand coming from the quarry of Missillac in France), authors [4] suggested two approaches (water content and fine content method/suction method) to predict the permanent deformation with different water contents and different fine contents. For the resilient behaviour, an improved Boyce–Hornych model [5] has shown that it has a good adaption to the changing anisotropy caused by the various water contents or fine contents under repeated loading. However, these findings were obtained just based on only one granular material—Missillac sand and the further verification with other materials for the general equations was greatly required.

As a result, in present study, a new material, UGM Bréfauchet, which is used as base layer material in low traffic pavements for full-scale tests at IFSTTAR in France, will be tested with repeated load triaxial tests (RLTT) at various water contents to describe accurately the permanent and resilient deformation behaviour. Then, based on the test results, the models or equations suggested for Missillac sand could be extended or checked.

Besides, the large UGM particles bring difficulties to fix the Hall-Effect strain measurement sensors, which is an accurate measurement method, on the triaxial sample. As a result, the actuator LVDT, external LVDT or internal LVDT are often applied in RLTTs to replace the Hall effect sensors [14,15]. However, Zhalehjoo et al., [16] showed significantly different *Mr* values with actuator LVDT, external LVDT, internal LVDT and Hall effect sensors based on a comprehensive comparative study. As a consequence, in this study, two technics for fixing the Hall effect sensors in the UGM for RLTTs will be proposed and compared with each other.

The objective of this work is to verify the evolution mechanism of permanent and resilient deformation (based on a subgrade sand) with a coarser UGM. Once the relationships can be reproduced, the results will be very meaningful to understand the evolution of UGM deformation in different layers in low traffic pavements. Finally, the findings will be helpful to reduce the number of tests required and to provide the parameters for pavement design.

## 2. Materials Studied

**Figure 1:**
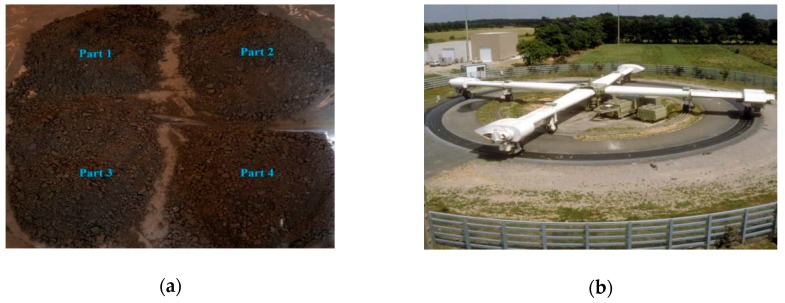
UGM Bréfauchet: (**a**) uniformizing the material; (**b**) IFSTTAR full-scale test track.

**Figure 2:**
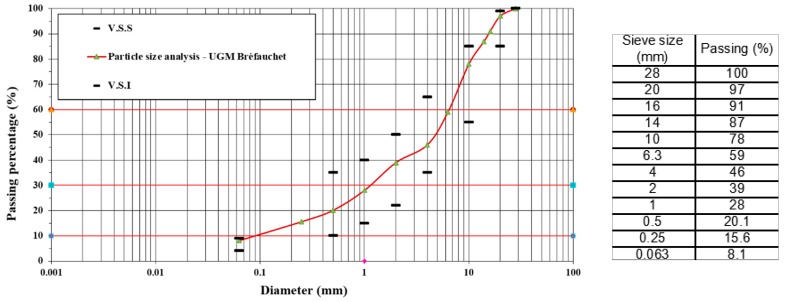
Particle size distribution.

The studied UGM Bréfauchet is a crushed gneiss aggregate from the site of Bréfauchet, near Nantes, France, as shown in Figure 1a. This material is used as a base layer material in low traffic pavements for full-scale pavement tests (Figure 1b) at IFSTTAR (Institut Français des Sciences et Technologies des Transports, de l’Aménagement et des Réseaux) in Nantes, France. The particle size of the UGM varies between 0 and 28 mm and the grain size distribution curve is presented in Figure 2. To fit the experimental devices, before the tests, the grains larger than 20 mm are removed.

**Figure 3:**
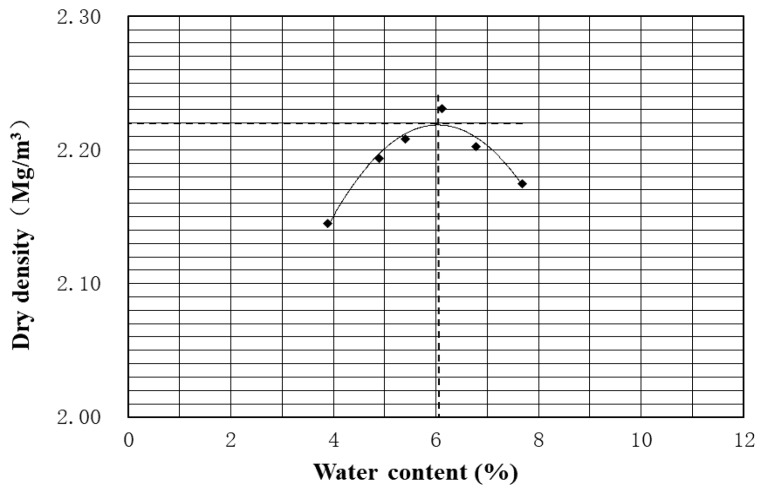
Modified Proctor compaction curve.

The modified Proctor test is completed with the automatic method [17]: the UGM is compacted in five layers in a CBR mold and the water content ranges from 3.8% to 7.8%. Figure 3 presents the modified Proctor compaction curve for UGM Bréfauchet. The optimum water content (OMC = 6.1%) and the maximum dry density (MDD = 2.218 Mg/m^3^) can be obtained based on the result.

In order to study the influence of water content on the mechanical behaviour of UGM Bréfauchet, the water content of triaxial test sample preparation is varied from 3% to 6% with a dry density of 2.13 Mg/m^3^ (96% of maximum dry density).

**Figure 4:**
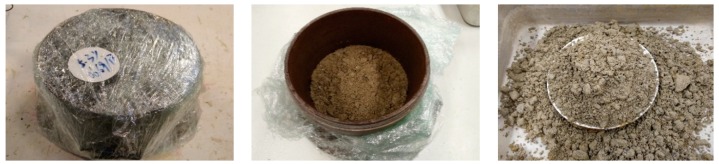
Filter paper method to measure matric suction.

As some researches mentioned above, the effect of water content is related to the suction in unsaturated soil. In fact, the suction corresponds to not only the moisture state but also to the microstructure of aggregates which can affect significantly the deformation. As a result, the SWRC of UGM Bréfauchet (wetting path) is investigated with the filter paper method [18]. The soil samples are compacted in two layers with a thickness of 3 cm per layer in a CBR mold and the Whatman no. 42 filter paper is placed between the two layers to measure the suction values of soil samples. The equipment and samples of suction measurement for UGM Bréfauchet are presented in Figure 4.

**Figure 5:**
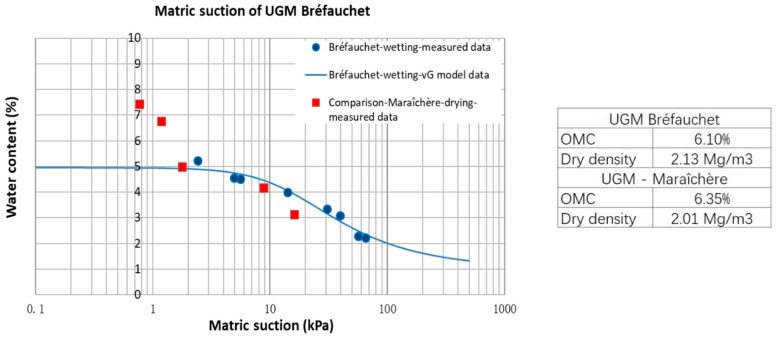
Matric suction of UGM Bréfauchet.

Figure 5 shows the variation of matric suction with water content in wetting path for UGM Bréfauchet. It can be stated that the matric suction is relatively low in UGM base layer (less than 100 kPa). The result is also compared with the suction for another UGM—Maraîchère in drying path [19]. The higher suction values for UGM Bréfauchet should be caused by a higher density prepared and a higher clay content for this material.

Besides, the van Genuchten model [20] is used to fit the measured data. The van Genuchten equation is expressed as

(1) $$w=w_{r}+\frac{\left(w_{s}-w_{r}\right)}{{\left[1+{\left(\alpha s\right)}^{n}\right]}^{m}},$$

where *w* is the actual soil water content at the suction *s* (kPa); *w_s_* (%) and *w_r_* (%) are the saturated water content and the residual water content; *α* is a parameter related to the air entry suction; *m* and *n* are the model parameters with the relationship: *m* = 1 − 1/*n*. Figure 5 also shows the model estimation which indicates that the van Genuchten model fits well with the measured values. The model parameters are summarized in Table 1.

It should be mentioned that, due to the time consuming and the limitation of device, two samples are tested for each studied water content. Finally, the test results of eight samples (lost two) from 2.2% to 5.2% are shown in Figure 5. This method has been calibrated with tensiometer method in the previous study [21].

## 3. Repeated Load Triaxial Test (RLTT)

RLTT is a common test to investigate the mechanical behaviour of granular materials. In the present study, it is used to simulate the in situ pavement conditions under cyclic loadings to describe the resilient deformation or permanent deformation of UGM at different initial hydraulic states.

### 3.1. Sample Preparation and Test Equipment

All the UGM Bréfauchet specimens are prepared, in the same way, before being subjected to triaxial tests. Specimens are prepared firstly by oven-drying for 24 h, then cool down aggregates are mixed with water using a large mixer to reach the target water contents. The wet materials are then stored in a well-sealed plastic bag for at least 24 h for moisture homogenization. Compaction is performed using a vibrating hammer following the French standard [22] in 7 layers for each specimen. All the tested specimens are prepared at a dry density of 2.13 Mg/m^3^ (96% of Maximum dry density). This is the maximum dry density which can be reached with this compaction method.

**Figure 6:**
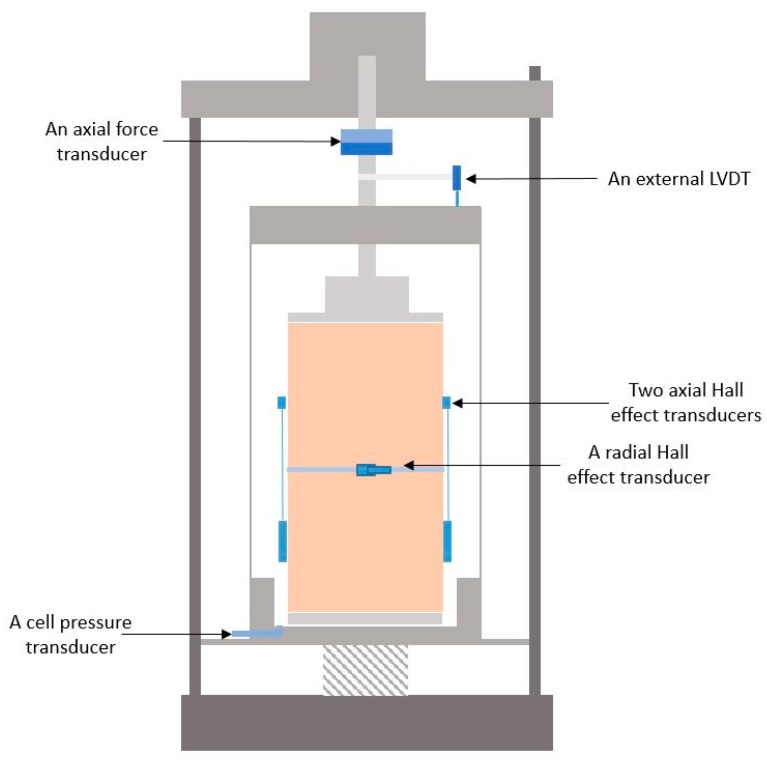
Schematic view of the triaxial cell placed in the loading frame and the transducers.

To test the UGM Bréfauchet with the largest particles whose diameter can reach 20 mm, a medium-scale triaxial device (Wykeham, Farrance), as shown in Figure 6 is used allowing testing specimens of 150 mm in diameter and 300 mm in height.

During a triaxial test, an axial stress (*σ*_1_) and a confining pressure (*σ*_3_) are applied on the specimen in the triaxial cell, simulating the loading state under pavement to measure the vertical strain (*σ*_1_) and the radial strain (*σ*_3_). Both the axial stress and the confining pressure are supplied by the pneumatic servo loading system.

The principal measurement system is a composite of stress/pressure transducers and displacement transducers. The typical transducers used in this study are:

- An axial force transducer;
- A cell pressure transducer;
- An external LVDT;
- Two inner Hall effect transducers (axial);
- An inner Hall effect transducer (radial).

As mentioned above, the axial deformation (*ε*_1_) and radial deformation (*ε*_3_) will be measured by the Hall effect transducers.

### 3.2. Monotonic Triaxial Test

To determine the shear strength parameters of the UGM Bréfauchet (friction angle and cohesion), monotonic undrained triaxial tests are firstly performed at water content of *w* = 4.4% and under different confining pressures/cell pressure *σ*_3_ (0 kPa, 7 kPa, 22kPa, 45 kPa). The shear test is controlled with a speed of 0.1%/min and terminated with 5% axial strain.

**Figure 7:**
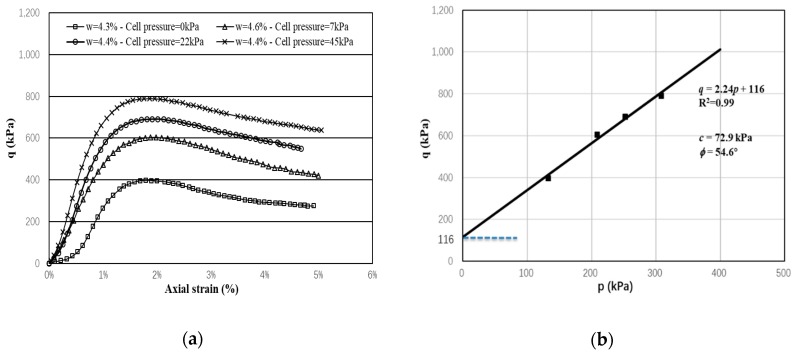
Monotonic test results (*w* = 4.4%): (**a**) *q*–*ε*_1_ curve; (**b**) strength parameters and rupture line. The mean normal stress *p* and deviator stress *q* are defined by.

Figure 7a presents the results of monotonic triaxial test. On the whole, with higher *σ*_3_, the peak deviator stress is more significant, as expected. The friction angle is 54.6° and the cohesion is 72.9 kPa. The rupture line, as shown in Figure 7b, will be helpful to determine the applied stress path and stress level in the following RLTTs.

(2) $$p=\frac{\sigma_{1}+2\sigma_{3}}{3},$$

and

(3) $$q=\sigma_{1}-\sigma_{3},$$

where, *σ*_1_ and *σ*_3_ are the major and minor principal stresses.

### 3.3. Two Methods for Fixing Displacement Transducers

In this study, the large particles (the diameter of grains can reach or be larger than 20 mm) bring difficulties to fix the three displacement transducers on the UGM sample and to have an accurate measurement and deform consistently with the sample, comparing with the sand samples [23].

To solve this problem, authors developed two sets of fixing devices for the displacement transducers to fit the larger particles.

**Figure 8:**
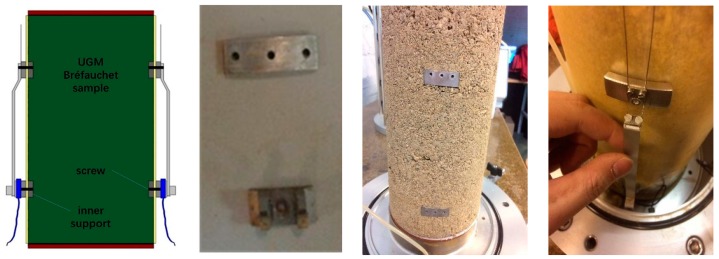
Fixing two vertical displacement transducers with screws.

For the first method (as shown in Figure 8), the pre-embedded metal bases and screws are used to fix the vertical displacement transducers. The radial displacement transducer is fixed by silica gel. This method can show basically implementation of strain measurement during repeated loading triaxial test. However, the thickness of 2 mm of the metal base is unfriendly for installation and it is difficult to follow the large deformation case. The contactless fixing of radial displacement transducer always generates an exorbitant radial strain in resilient test phase with a variable confining pressure. Besides, in order to prevent the intrusion of water from screw holes, silica glue is used carefully which is really hard to operate and fails often.

**Figure 9:**
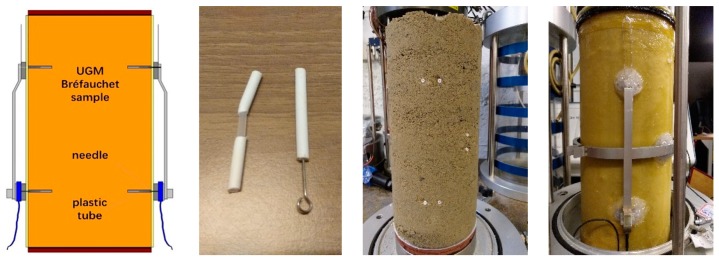
Fixing three displacement transducers with tubes.

As a consequence, another method is developed with tubes (as shown in Figure 9): the tubes, about 5 cm, are filled with silica gel and pre-embedded into specimen (about 2.5 cm) from the drilled hole on the compaction mold during the compaction. After compaction, the protrusion part of each tube is cut and removed before the de-molding. Then, the vertical and radial displacement transducers can be well fixed by the needles inserted into tube bases.

### 3.4. Test Procedures

**Figure 10:**
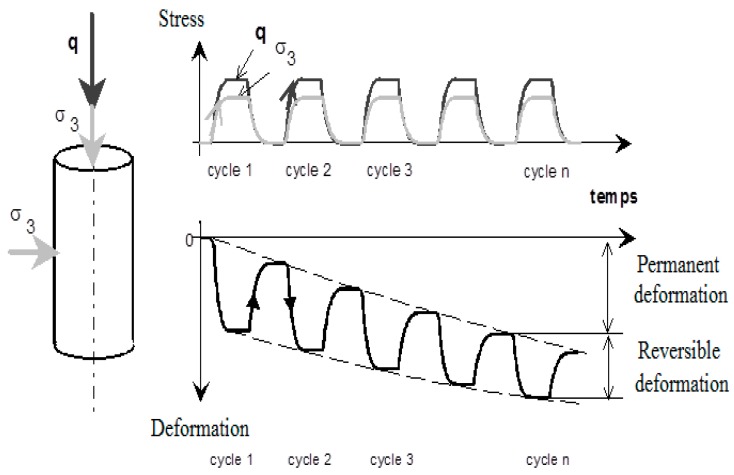
The principle of RLTT.

Figure 10 presents the principle of RLTT: under the repeated loading (*q* & *σ*_3_), the increase of deformation (axial/radial) can be divided into two parts in each cycle. The unrecoverable part is called permanent/plastic deformation, which accumulates gradually as number of cycles increasing. Another recoverable part is called reversible deformation. After adequate cycles, the increase of the unrecoverable part is quiet small. It means that the accumulated permanent deformation tends to stabilization and then the recoverable part can be treated as resilient deformation.

As a result, to study this complex cyclic behaviour of UGM Bréfauchet (permanent and resilient behaviour), a conditioning phase under CCP (constant confining pressure) loading to stabilize the accumulation of permanent strains and a following resilient test phase under VCP (variable confining pressure) loading to investigate the non-linear elastic behaviour are applied on a specimen.

- For the conditioning phase, the cyclic stress path of Δ*q*/Δ*p* = 3 (Δ*p* = 166.67 kPa, Δ*q* = 500 kPa) from an initial stress state (*p*_0_ = 70 kPa, *q*_0_ = 0 kPa) is applied at a frequency of *f* = 0.5 Hz. The number of cycles is chosen as *N* = 20,000 cycles. When the permanent deformation rate Δ*ε*_1_^p^/Δ*N* is lower than 1 × 10^−7^, we can state that the permanent deformation achieves the equilibrium state [24].
- For the following resilient phase, five stress paths (Δ*q*/Δ*p* = 0; 1; 1.5; 2; 2.5) are applied on the same sample in sequence from an initial stress state (*p*_0_ = 20 kPa, *q*_0_ = 0 kPa). Each stress path contains 100 loading and unloading cycles, with a frequency of *f* = 0.05 Hz.

**Figure 11:**
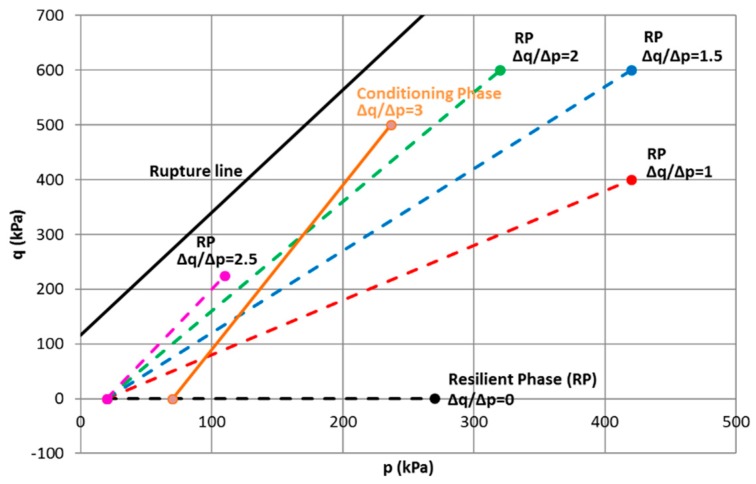
Stress paths used in conditioning phase and resilient test phase.

Figure 11 and Table 2 summarize the stress paths used in conditioning phase and resilient test phase.

## 4. Results and Discussions

### 4.1. Permanent Deformation

**Figure 12:**
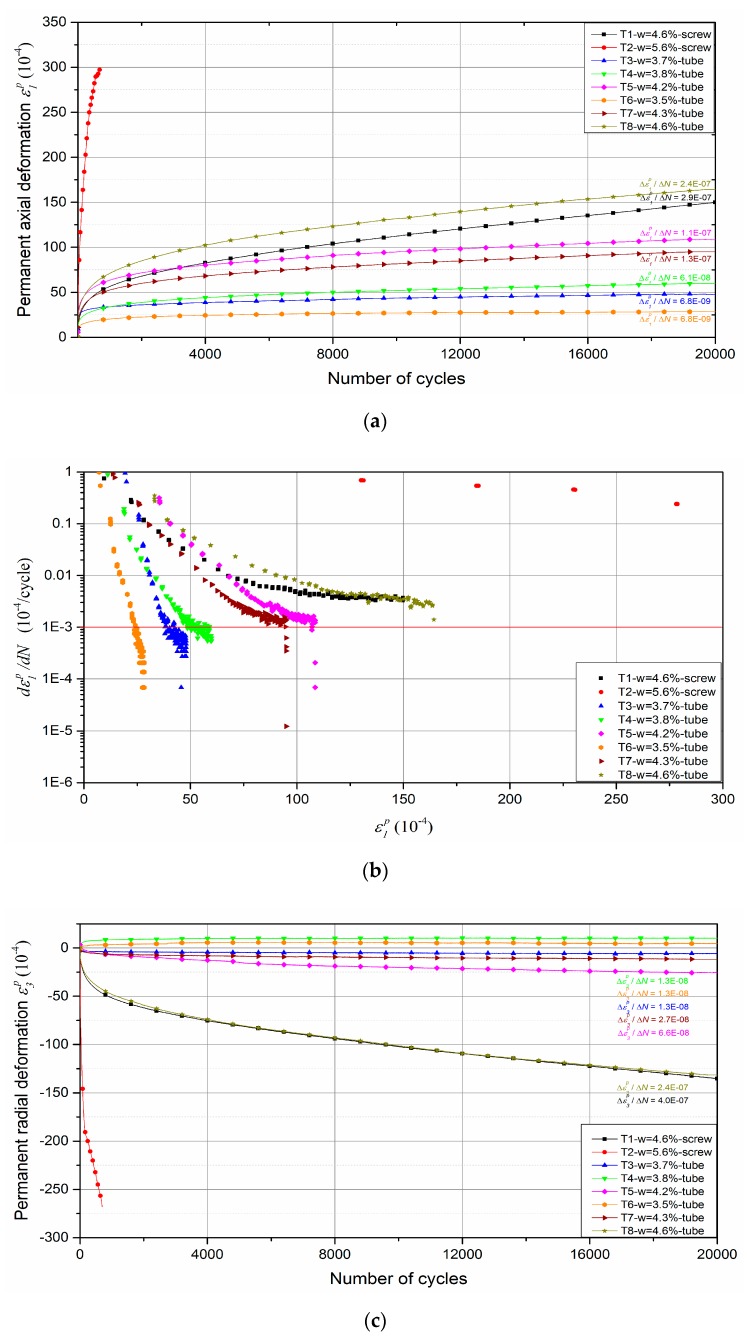
Permanent deformation behaviour: (**a**) Permanent axial deformation versus number of cycles; (**b**) Permanent axial deformation rate versus the accumulated permanent axial deformation; (**c**) Permanent radial deformation versus number of cycles.

The results of conditioning phase are used to determine the permanent deformation behaviour. It can be stated that the permanent axial deformation *ε*_1_^p^ increases with an increase of the number of cycles at different water contents as illustrated in Figure 12a. On the whole, the higher the water content, the larger the permanent axial deformation. For most water contents, the permanent axial strain increases quickly during the first several cycles and then tends to be constant with a low increment for each loading cycle. In particular, at water content of *w* = 5.6%, the sample is rapidly destroyed under the repeated loading. The evolution of permanent axial deformation rate versus the accumulated permanent axial deformation at various water contents is also presented in Figure 12b. It can be stated that the permanent axial deformation rate decreases rapidly with an increase of permanent axial deformation when the water content is lower than 4.3%. Finally, the permanent axial deformation rates (Δ*ε*_1_*^p^*/Δ*N*) at the end of each test are less than or close to 10^−7^/cycle, except at water content of 5.6%. As a result, we can state that the permanent axial deformation has achieved the equilibrium state after 20,000 cycles and the following resilient phase can be applied on the samples.

For permanent radial deformation, the results show that the *ε*_3_^p^ also increases with the number of cycles at different water contents as shown in Figure 12c. The effect of water content on the permanent radial deformation is the same as permanent axial deformation: generally, the higher the water content, the larger the permanent radial deformation. Based on the value of Δ*ε*_3_^p^/Δ*N* at the end of each test, it can be also stated that the permanent radial deformation at end of each test achieves the equilibrium state after 20,000 cycles.

Besides, based on the results of T1-*w* = 4.6%-screw and T8-*w* = 4.6%-tube, it can be also stated that the effect of different methods of fixing displacement transducers on the permanent deformation behaviour: for the permanent axial deformation, tube method has 20 × 10^−4^ deformation larger than screw method; for the permanent radial deformation, these two methods accord with each other, which can be explained with the constant confining pressure (CCP) in conditioning phase: the radial sensor is always pressured on the sample by the constant confining pressure for both methods, the difference of radial deformation between both methods cannot be observed.

### 4.2. Resilient Deformation

In this study, the resilient deformation behaviour is described by the resilient volumetric deformation (*ε_v_^r^*) and the resilient deviatoric deformation (*ε_q_^r^*) defined as

(4) $$\varepsilon_{v}^{r}=\varepsilon_{1}^{r}+2\varepsilon_{3}^{r}$$

(5) $$\varepsilon_{q}^{r}=\frac{2\left(\varepsilon_{1}^{r}-\varepsilon_{3}^{r}\right)}{3}$$

where, *ε*_1_*^r^* is the resilient axial deformation and *ε*_3_*^r^* is the resilient radial deformation.

**Figure 13:**
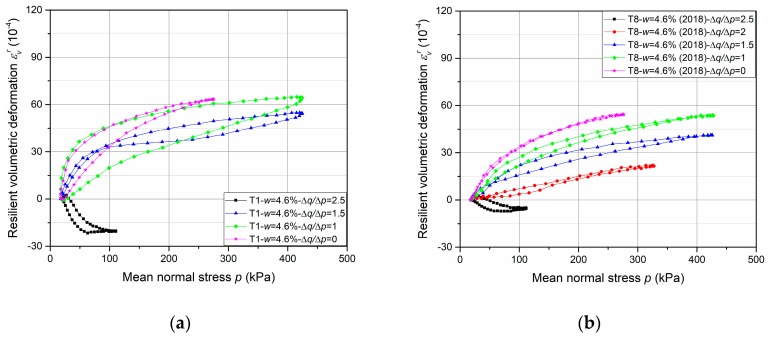
Resilient volumetric deformation *ε*_v_^r^ with different fixing methods: (**a**) T1-*w* = 4.6%-screw; (**b**) T8-*w* = 4.6%-tube.

**Figure 14:**
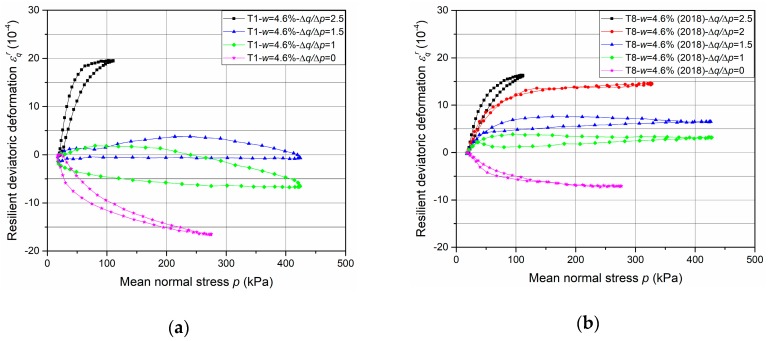
Resilient deviatoric deformation *ε*_q_^r^ with different fixing methods: (**a**) T1-*w* = 4.6%-screw; (**b**) T8-*w* = 4.6%-tube.

**Figure 15:**
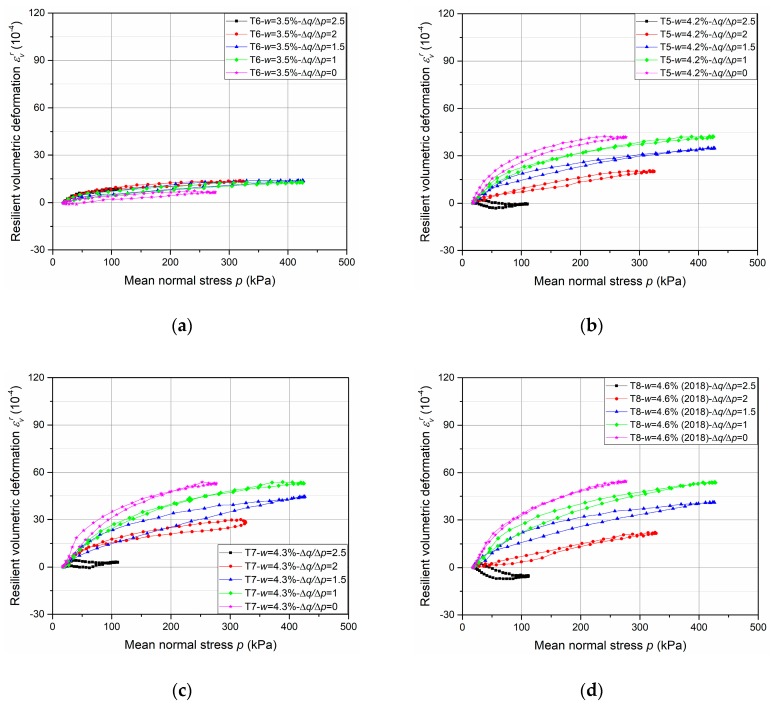
Resilient volumetric deformation *ε_v_^r^* at different water contents: (**a**) T6-*w* = 3.5%; (**b**) T5-*w* = 4.2%; (**c**) T7-*w* = 4.3%; (**d**) T8-*w* = 4.6%.

**Figure 16:**
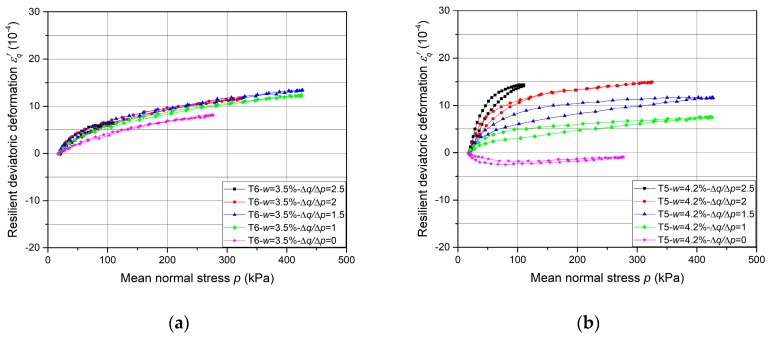

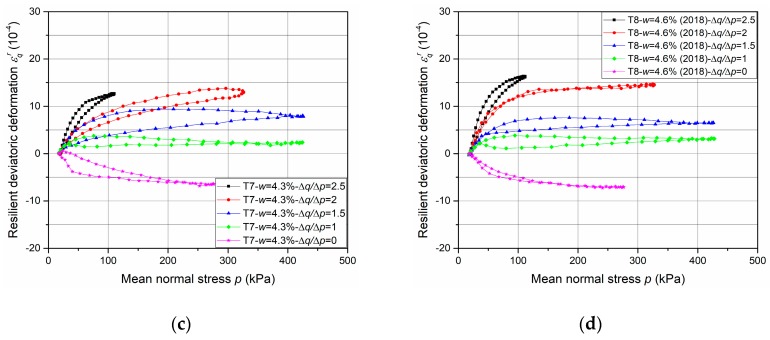
Resilient deviatoric deformation ε_q_^r^ at different water contents: (**a**) T6-*w* = 3.5%; (**b**) T5-*w* = 4.2%; (**c**) T7-*w* = 4.3%; (**d**) T8-*w* = 4.6%.

The results of resilient test phase are presented in Figure 13, Figure 14, Figure 15 and Figure 16.

First of all, the comparison of resilient volumetric and deviatoric deformation between two fixing methods are introduced in Figure 13 and Figure 14 respectively, based on the results of T1-*w* = 4.6%-screw and T8-*w* = 4.6%-tube. It can be stated that, for the resilient volumetric deformation, the screw method has a larger value than the tube method and the unsmooth curves with large open loops suggest the poorly accurate measurement with the screw method. The same phenomenon can be observed for resilient deviatoric deformation.

The results can be explained with the inaccurate measurement of *ε*_3_, which is the most likely to be affected by the variable confining pressure. Besides, the influence of deformation development on axial deformation measurement is not presented clearly with the present results which have no large deformation. Normally, only 2 mm thickness for screw bases leads to a sharp increase of deformation or sensors failure when a large deformation and an incline of screw base occur. Consequently, the method with tubes is suggested to apply mainly in this study and in the future based on the accurate measurement and good adaptability during the RLTT.

Figure 15 and Figure 16 show the resilient volumetric deformation *ε*_v_^r^ and the resilient deviatoric deformation *ε*_q_^r^, respectively, that are obtained for different stress paths (Δ*q*/Δ*p* = 0; 1; 1.5; 2; 2.5) at different water contents (*w* = 3.5%, 4.2%, 4.3% and 4.6%, as examples, with tube method). Based on the results, following observations can be stated:

- The cycles show that the behaviour of the UGM is nonlinear, and depends on the mean stress *p* and the stress ratio Δ*q*/Δ*p*. It can be stated that *ε_q_^r^* increases while *ε_v_^r^* decreases with the ratio Δ*q*/Δ*p* increases.
- The effect of water content on resilient deformations is observed: *ε_v_^r^* and *ε_q_^r^* increase with the water content and present the largest values at the water content (4.6%) in all stress paths nearly.
- The very slight hysteresis for the cycles suggests that the behaviour is close to pure elasticity and the measurements with tube method is more accurate enough compared to that with the metallic inserts/screws.

**Figure 17:**
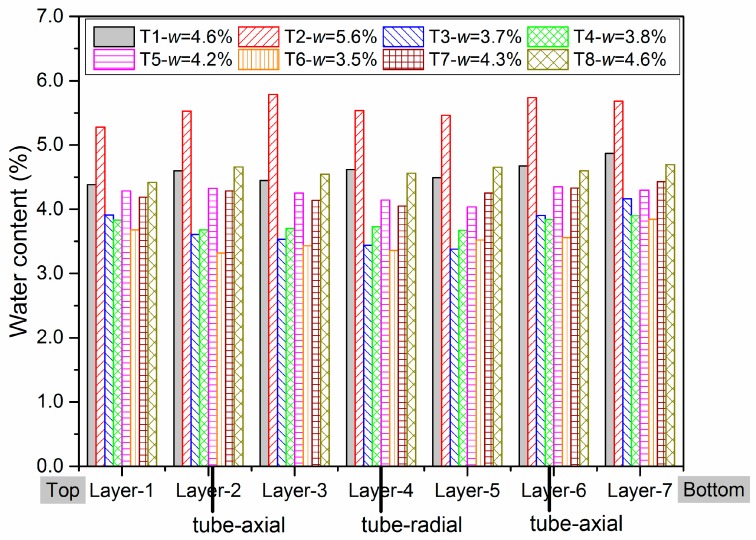
Water distribution in seven layers in each sample.

After tests, all the specimens are cut into seven equal pieces to measure the water content and the water distribution. As shown in Figure 17, the results present the water contents for different pieces in different samples after testing phases. It can be also stated that all the samples have a homogeneous water content distribution and both tube and screw methods with a water proof silicone well spread, can prevent the intrusion of water effectively.

## 5. Modelling of Permanent and Resilient Behaviour

In this section, the proposed/modified models for permanent behaviour [4] and resilient behaviour [5] based on the researches for Missillac sand will be verified with the test results (RLTT and SWRC) of UGM Bréfauchet as follows.

### 5.1. Determination of Permanent Axial Deformation with Water Content and Suction

As mentioned above, the prediction models of permanent axial deformation proposed in previous research [4] are available to capture the evolution of permanent behaviour taking into account the number of cycles, the stress level, the water content, and the fine content of the granular material. Two approaches are used: one is based on the water contents and fine contents and the other is based on suction values and both approaches show good capacities. In this study, the fine content and stress level are not considered. As a result, the models used for UGM Bréfauchet are rewritten as:

Based on water content:

(6) $$\varepsilon_{1}^{p}=a\cdot {\left(w/v\right)}^{o}\cdot \left(1-{\left(N/N_{0}\right)}^{\left(v^{\prime }/w\right)}\right),$$

where, *ε*_1_*^p^* is permanent axial deformation; *w* is water content; *N* is number of cycles; *N*_0_ is number of cycles before the first measurement; a, *v*, o and *v*’ are parameters.

Based on suction:

(7) $$\varepsilon_{1}^{p}=b\cdot {\left(S/S^{*}\right)}^{d}\cdot \left(1-{\left(N/N_{0}\right)}^{e\cdot \ln\left(S/S_{a}\right)+f}\right),$$

where, *S* is suction value; S* is suction value corresponding to the intersection point of wetting and drying paths; *b*, *d*, *e*, and *f* are parameters; *S*_a_ is equal to 100kPa. In this study, the S* is estimated in a range from 1.5kPa to 3.5 kPa for the lack of drying path data.

**Figure 18:**
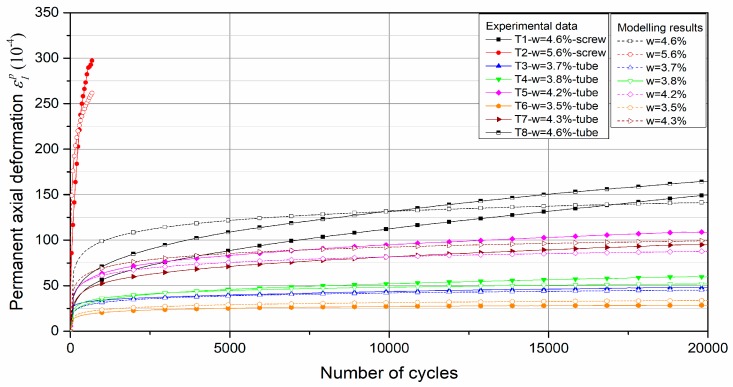
Test results, as well as the model prediction, for *ε*_1_^p^ based on water content.

**Figure 19:**
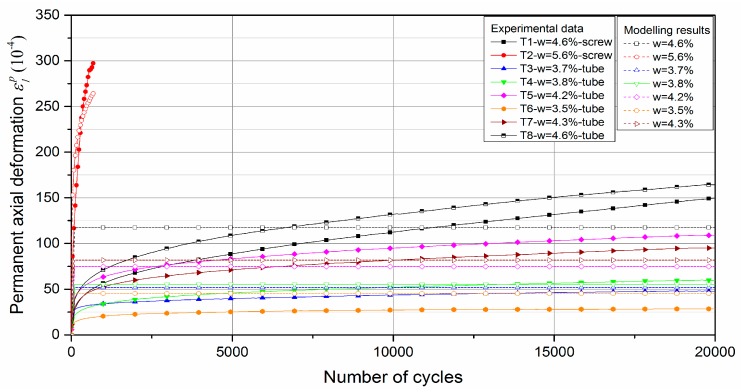
Test results, as well as the model prediction, for *ε*_1_^p^ based on suction (S* = 2.5 kPa).

Figure 18 and Figure 19 show the modelling prediction compared with test results with water content based model and suction based model respectively. The parameters of the models are presented in Table 3 and Table 4. Based on the results, it can be stated that the proposed models for the Missillac sand, a granular material, used for the subgrade layer, give satisfactory results for the UGM Bréfauchet used for the base layer with coarser particles.

Besides, it is obvious that the water content based model can describe the permanent deformation behaviour and its evolution more accurately than suction based model. The phenomenon can be explained as follows, for the granular materials, the matric suction value and the influence of suction on mechanical behaviour decrease with the increase of coarse particles. In this study, the suction of UGM Bréfauchet is less than 70 kPa with a reasonable water content range, which is far less than the maximum applied loads during the RLTT. Besides, as we mentioned in the former study for Missillac sand [4,13], the suction corresponds not only to the saturation state but also to the fine content and further the microstructure in aggregates, which influences significantly the deformation behaviour of granular materials under repeated loads. However, for UGM Bréfauchet, the mechanical behaviour is affected gradually by the coarse particle structures which cannot be evaluated by suction.

It can be concluded that the models proposed for granular materials have a good capacity to capture the general trend of the evolution of the permanent deformation with the number of cycles. The modelling accuracy is decreasing gradually with increasing particle size with suction based model.

### 5.2. Determination of Resilient Deformation with Modified Boyce Model

Boyce (1980) [25] proposed an isotropic non-linear model for predicting the resilient deformation behaviour of granular materials. Then, Hornych and co-workers [26] introduced the anisotropic response of granular materials into Boyce model through multiplying the axial stress by an anisotropy coefficient γ_1_. The modified model was expressed as

(8) $$\varepsilon_{v}^{r}=\frac{p^{*}{}^{n}}{p_{a}{}^{n-1}}\left[\frac{\gamma_{1}+2}{3K_{a}}+\frac{n-1}{18G_{a}}\left(\gamma_{1}+2\right)\cdot {\left(\frac{q^{*}}{p^{*}}\right)}^{2}+\frac{\gamma_{1}-1}{3G_{a}}\cdot \frac{q^{*}}{p^{*}}\right],$$

(9) $$\varepsilon_{q}^{r}=\frac{2}{3}\cdot \frac{p^{*}{}^{n}}{p_{a}{}^{n-1}}\left[\frac{\gamma_{1}-1}{3K_{a}}+\frac{n-1}{18G_{a}}\left(\gamma_{1}-1\right).{\left(\frac{q^{*}}{p^{*}}\right)}^{2}+\frac{2\cdot \gamma_{1}+1}{6G_{a}}\cdot \frac{q^{*}}{p^{*}}\right],$$

(10) $$p^{*}=\frac{\gamma_{1}\sigma_{1}+2\sigma_{3}}{3},$$

(11) $$q^{*}=\gamma_{1}\sigma_{1}-\sigma_{3},\;0<\gamma_{1}<1,$$

where, *K*a, *G*a, and *n* are parameters; *γ*_1_ is the axial anisotropy coefficient, *γ*_1_ = |*ε*_1_/*ε*_3_|; *P*a is 100 kPa.

Based on the results of Missillac sand, the radial anisotropy coefficient *γ*_3_ was suggested by authors [5] to replace the axial anisotropy coefficient *γ*_1_ to fit the changing anisotropy behaviour of granular materials under the repeated loading. The improved model was expressed as

(12) $$\varepsilon_{v}^{r}=\frac{p^{*}{}^{n}}{p_{a}{}^{n-1}}\left[\frac{1+2\gamma_{3}}{3K_{a}}+\frac{n-1}{18G_{a}}\left(1+2\gamma_{3}\right).{\left(\frac{q^{*}}{p^{*}}\right)}^{2}+\frac{1-\gamma_{3}}{3G_{a}}\cdot \frac{q^{*}}{p^{*}}\right],$$

(13) $$\varepsilon_{q}^{r}=\frac{2}{3}\cdot \frac{p^{*}{}^{n}}{p_{a}{}^{n-1}}\left[\frac{1-\gamma_{3}}{3K_{a}}+\frac{n-1}{18G_{a}}\left(1-\gamma_{3}\right)\cdot {\left(\frac{q^{*}}{p^{*}}\right)}^{2}+\frac{2+\gamma_{3}}{6G_{a}}\cdot \frac{q^{*}}{p^{*}}\right],$$

(14) $$p^{*}=\frac{\sigma_{1}+2\gamma_{3}\sigma_{3}}{3},$$

(15) $$q^{*}=\sigma_{1}-\gamma_{3}\sigma_{3},\;\gamma_{3}>1,$$

where, *γ_3_* is the radial anisotropy coefficient, *γ*_3_ = |*ε*_3_/*ε*_1_|.

In this section, based on the resilient test results of UGM Bréfauchet as shown in Figure 15 and Figure 16, the modelling results of these two sets of equations will be compared to check the model improvement with radial anisotropy coefficient *γ*_3_.

**Figure 20:**
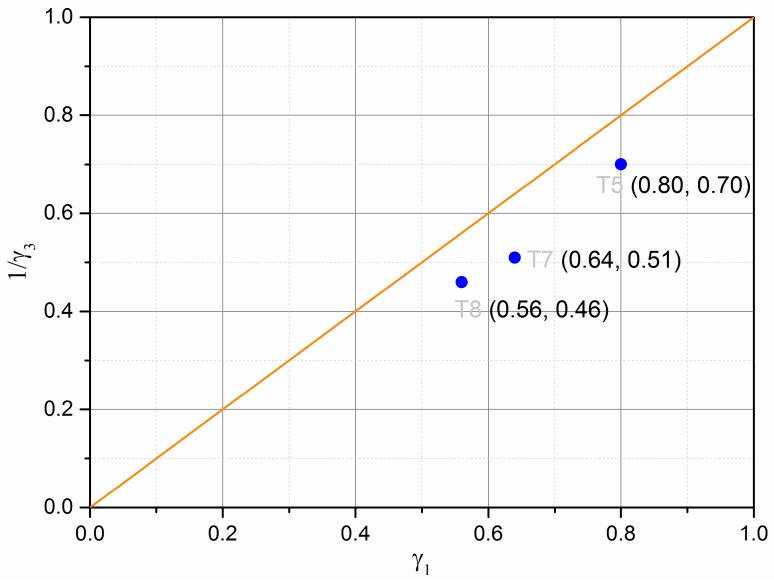
Relationships between γ_1_ and 1/γ_3._

The parameter optimization of Equations (8)–(11) for UGM Bréfauchet with *γ*_1_ is presented in Table 5 and the parameter optimization of Equations (12)–(15) for UGM Bréfauchet with *γ*_3_ is presented in Table 6. Based on the result of T5-*w* = 4.2%, T7-*w* = 4.3%, and T8-*w* = 4.6%, it can be stated that both sets of equations have good correlation coefficients for the resilient behaviour and the Equations (12)–(15) show a more accurate modelling result. The curves of T6-*w* = 3.5%, in different stress paths, are close together as shown in Figure 15a and Figure 16a. As a result, the optimized parameters with both sets of equations are very poor and cannot be used for the comparison in this section. Besides, Figure 20 shows the relationships between *γ*_1_ and 1/*γ*_3_. It can be confirmed that the reciprocal relationship between *γ*_1_ and *γ*_3_ for UGM can change under a repeated loading as observed for Missillac sand [5]. In conclusion, the resilient test results of UGM Bréfauchet can verify the improved Boyce–Hornych model (Equations (12)–(15)) which is accurate and general for the unbound granular materials.

**Figure 21:**
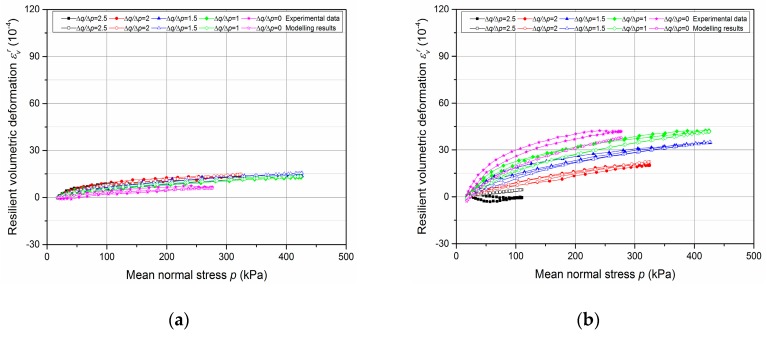

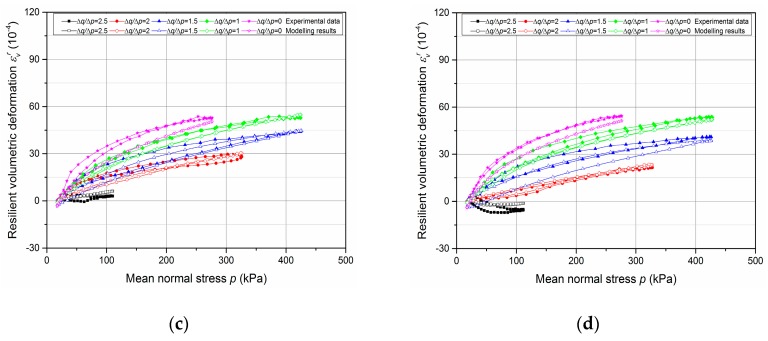
Modelling of *ε*_v_^r^ based on Equations (12)–(15) for UGM Bréfauchet at different water content: (**a**) T6-*w* = 3.5%; (**b**) T5-*w* = 4.2%; (**c**) T7-*w* = 4.3%; (**d**) T8-*w* = 4.6%.

**Figure 22:**
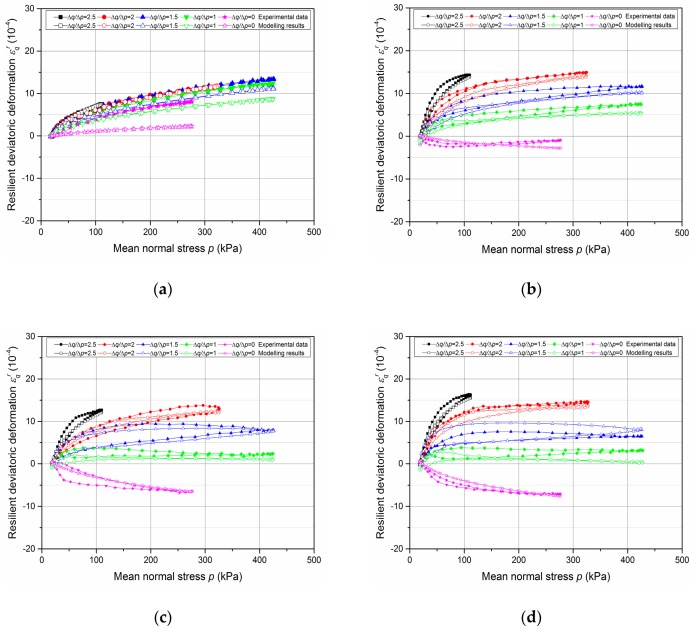
Modelling of *ε*_q_^r^ based on Equations (12)–(15) for UGM Bréfauchet at different water contents: (**a**) T6-*w* = 3.5%; (**b**) T5-*w* = 4.2%; (**c**) T7-*w* = 4.3%; (**d**) T8-*w* = 4.6%.

Figure 21 and Figure 22 show the modelling of *ε_v_^r^* and *ε_q_^r^* values for UGM Bréfauchet at different water contents based on Equations (12)–(15). As described by correlation coefficients above, the very good estimated results (both *ε_v_^r^* and *ε_q_^r^*) can be obtained.

## 6. Conclusions

Granular layers play an important role in the overall performance of the structure, especially for the bearing capacity. In the previous studies, the performance of subgrade granular material— Missillac sand has been well analyzed and a series of prediction models was proposed or modified to describe the permanent and resilient behaviour at different water contents and different fine contents. In the present work, a much coarser UGM Bréfauchet, which is used as base layer material in low traffic pavements for full-scale pavement tests, has been studied to check the evolution mechanism. Based on the investigation of these two granular materials, it can be concluded that:

- Both approaches based on water content/suction have a good capacity to describe the permanent deformation behaviour of UGM at different water contents. However, the modelling accuracy is decreasing gradually with increasing particle size with suction-based model.
- For the resilient behaviour of UGM, the improved Boyce–Hornych model has a good ability to capture the changing anisotropy caused by the various water contents or fine contents under repeated loading.
- The two methods of fixing the sensors on the materials with large particles for RLTTs are proposed and compared with each other. The results suggest that the tube method is more suitable for the large particle UGM as the accurate measurement and good adaptability during the RLTT.

Consequently, this study is helpful to understand the evolution of permanent and resilient deformation of a wide range of different granular materials. The models can be used for other similar granular materials directly and reduce the number of tests required to predict the deformation behaviour.

It should be also mentioned that, due to the time consuming and the complexity of the operation, the number of the test in this study is not so abundant. As a result, for the future work, plenty of repetition tests will be continued for further verification and statistical analysis. Besides, further numerical modelling will be also conducted.

## Figures and Tables

**Table 1:** Parameters of van Genuchten model.

**UGM Bréfauchet Wetting path**	**Parameters of VG model**	***α***	***n***	***m***	***w_s_* (%)**	***w_r_*(%)**
		0.199	1.932	0.482	5.4	2.9

**Table 2:** Stress paths used in conditioning phase and resilient test phase.

Materials	Δ*q*/Δ*p*	Δ*p* (kPa)	Δ*q* (kPa)	Confining Pressure (kPa)
UGM Bréfauchet	3(Conditioning)	166.67	500	70–CCP
	0	250	0	270–VCP
	1	400	400	286.67–VCP
	1.5	400	600	220–VCP
	2	300	600	120–VCP
	2.5	90	225	35–VCP

**Table 3:** Parameters of the model based on water content.

**UGM Bréfauchet**	***a***	v	***o***	$v^{\prime }$	**R^2^**
	69.644	1.277	6.246	−0.0003	0.937

**Table 4:** Parameters of the model based on suction.

UGM Bréfauchet	S^*^(kPa)	***b***	***d***	***e***	***f***	**R^2^**
	1.5	351.627	−0.733	−1.393	−9.642	0.891
	2.5	267.284	−0.800	−0.464	−3.218	0.896
	3.5	219.425	−0.867	−0.677	−4.687	0.898

**Table 5:** Parameter optimization of Equations (8)–(11) for UGM Bréfauchet with *γ*_1_.

*w* (%)	Δ*q*/Δ*p*	Parameters (UGM Bréfauchet)				Ccorrel
		*Ka*	*Ga*	*n*	*γ*_1_	
3.5	0; 1; 1.5; 2; 2.5	152.63	118.54	0.02	29.07	0.636
4.2	0; 1; 1.5; 2; 2.5	16.77	38.47	0.27	0.80	0.799
4.3	0; 1; 1.5; 2; 2.5	14.15	32.06	0.33	0.64	0.835
4.6	0; 1; 1.5; 2; 2.5	9.04	23.42	0.20	0.56	0.827
Average values		48.15	53.12	0.21	7.77	/

**Table 6:** Parameter optimization of Equations (12)–(15) for UGM Bréfauchet with *γ_3_*.

*w* (%)	Δ*q*/Δ*p*	Parameters (UGM Bréfauchet)				Ccorrel
		*Ka*	*Ga*	*n*	*γ*_3_	
3.5	0; 1; 1.5; 2; 2.5	87.02	70.90	0.45	0.20	0.573
4.2	0; 1; 1.5; 2; 2.5	23.55	54.18	0.29	1.43	0.821
4.3	0; 1; 1.5; 2; 2.5	25.80	65.39	0.33	1.96	0.853
4.6	0; 1; 1.5; 2; 2.5	18.62	55.91	0.22	2.18	0.865
Average values		38.75	61.60	0.32	1.44	/
